# Association of the Single Nucleotide Polymorphisms rs11556218, rs4778889, rs4072111, and rs1131445 of the Interleukin-16 Gene with Ovarian Cancer

**DOI:** 10.3390/ijms251910272

**Published:** 2024-09-24

**Authors:** Rafał Watrowski, Eva Schuster, Toon Van Gorp, Gerda Hofstetter, Michael B. Fischer, Sven Mahner, Stefan Polterauer, Robert Zeillinger, Eva Obermayr

**Affiliations:** 1 Department of Obstetrics and Gynecology, Helios Hospital Muellheim, Teaching Hospital of the University of Freiburg, Heliosweg 1, 79379 Muellheim, Germany; rafal.watrowski@gmx.at; 2 Faculty of Medicine, University of Freiburg, 79106 Freiburg, Germany; 3 Molecular Oncology Group, Department of Obstetrics and Gynecology, Comprehensive Cancer Center-Gynecologic Cancer Unit, Medical University of Vienna, Waehringer Guertel 18-20, 1090 Vienna, Austria; eva.schuster@meduniwien.ac.at (E.S.); stefan.polterauer@muv.ac.at (S.P.); robert.zeillinger@muv.ac.at (R.Z.); 4 Division of Gynecologic Oncology, University Hospital Leuven, 3000 Leuven, Belgium; toon.vangorp@uzleuven.be; 5 Leuven Cancer Institute, Catholic University of Leuven, 3000 Leuven, Belgium; 6 Department of Pathology, Medical University of Vienna, Waehringer Guertel 18-20, 1090 Vienna, Austria; gerda.hofstetter@meduniwien.ac.at; 7 Department of Blood Group Serology and Transfusion Medicine, Medical University of Vienna, Waehringer Guertel 18-20, 1090 Vienna, Austria; michael.b.fischer@meduniwien.ac.at; 8 Center for Biomedical Technology, Department for Biomedical Research, Danube University Krems, Dr.-Karl-Dorrek-Straße 30, 3500 Krems, Austria; 9 Department of Gynecology, University Medical Center Hamburg-Eppendorf, 20246 Hamburg, Germany; sven.mahner@med.uni-muenchen.de; 10 Department of Obstetrics and Gynecology, University Hospital, Ludwig-Maximilians-University Munich, 81377 Munich, Germany

**Keywords:** ovarian cancer, interleukin-16, IL-16, single nuclear polymorphism, cancer risk, premenopausal cancer

## Abstract

Single nucleotide polymorphisms (SNPs) of the IL-16 gene have been reported to influence the risk of several cancers, but their role in ovarian cancer (OC) has not been studied. Using the restriction fragment length polymorphism (PCR-RFLP) method, we examined four IL-16 SNPs: rs11556218 (T > G), rs4778889 (T > C), rs4072111 (C > T), and rs1131445 (T > C) in blood samples from 413 women of Central European descent, including 200 OC patients and 213 healthy controls. Among the patients, 62% were postmenopausal, 84.5% were diagnosed in late stages (FIGO IIb-IV), and 73.5% had high-grade serous OC (HGSOC). Minor allele frequencies in controls were 9.2% for rs11556218 (G allele), 13.7% for rs4778889 (C allele), 10.4% for rs4072111 (T allele), and 32.3% for rs1131445 (C allele). We found significant associations of rs11556218 (G vs. T allele: OR 2.76, 95% CI 1.84–4.14, *p* < 0.0001) with elevated OC risk in the whole cohort (*p* < 0.001) and in both premenopausal (*p* < 0.001) and postmenopausal (*p* = 0.001) subgroups. These associations remained significant across heterozygote (*p* < 0.001), dominant (*p* < 0.001), and overdominant (*p* < 0.001) models. IL-16 rs4778889 was associated with OC risk predominantly in premenopausal women (*p* < 0.0001 in almost all models). In the whole cohort, the C allele was associated with OC risk (OR 1.54, CI 95% 1.06–2.23, *p* = 0.024), and the association of rs4778889 was significant in dominant (*p* = 0.019), overdominant (*p* = 0.033), and heterozygote (*p* = 0.027) models. Furthermore, rs4778889 was linked with HGSOC (*p* = 0.036) and endometriosis-related OC subtypes (*p* = 0.002). No significant associations were found for rs4072111 or rs1131445 (*p* = 0.81 or 0.47, respectively). In conclusion, rs11556218 and rs4778889 SNPs are associated with OC risk, especially in premenopausal women.

## 1. Introduction

Ovarian cancer (OC), affecting 1.1–1.5% of women during their lifespan, is the most lethal among all gynecological malignancies [1,2,3]. In 2019, OC accounted for 3.4% of new cancer diagnoses and 4.7% of cancer-related deaths worldwide, translating to 300,000 new cases and 200,000 deaths [1]. In Europe, OC is the fifth most common cause of cancer death [3]. Globally, the incidence, related deaths, and disability-adjusted life-years of OC doubled between 1990 and 2020 [2].

Most ovarian tumors originate from ovarian or tubal epithelium (90%), with stromal cells (5–6%) and germ cells (2–3%) as less common origins [4]. Due to their similar biology and spread patterns, the term OC includes carcinomas from the ovary, fallopian tube, and peritoneum [5]. High-grade serous OC (HGSOC), arising from intraepithelial tubal precursor lesions, is the most common and aggressive subtype, making up about 75% of cases [5,6]. Endometrioid and clear cell OC subtypes, often developing from endometriotic lesions, have a comparatively better prognosis and are classified as endometriosis-related OC (EROC) subtypes [7,8]. The prognosis for OC remains poor, with 5-year survival rates between 40% and 50%, largely due to the cancer’s biological aggressiveness, frequent acquired chemotherapy resistance, and late-stage diagnosis (FIGO stage III or IV) [1,2,3]. Early OC stages, which have better outcomes, often evade detection due to non-specific symptoms and limited biomarker sensitivity and specificity [3,9,10,11,12]. OC treatment typically involves extensive cytoreductive surgery, ideally primary and complete, followed by or preceding chemotherapy and targeted therapies [3,13,14,15].

Genetic factors contribute to OC in at least 25% of cases [16,17]. Alterations in BRCA1 and BRCA2 genes account for over half of hereditary cases, while mutations in TP53, BARD1, CHEK2, RAD51, and PALB2 contribute to at least 15% of cases [16,17,18]. Besides high and moderate penetrance genes, genome-wide association studies, candidate gene studies, and next-generation sequencing have identified common alleles with low penetrance susceptibility [16,19]. Single nucleotide polymorphisms (SNPs), the most common genetic variations, result from a single nucleotide (A, T, C, or G) substitution in coding or non-coding DNA. Even minimal changes in genes regulating the cell cycle, DNA repair, immune response, angiogenesis, or metabolic pathways can influence carcinogenesis [20]. To date, around 100 SNPs have been proposed as factors potentially affecting OC risk [21].

Inflammation and inflammatory mediators play a critical role in the development, progression, metastasis, and chemoresistance of OC [22,23,24]. The communication between cells involved in the immune response occurs through cytokines (secreted messenger molecules). Among them, interleukins are named for their expression by leukocytes and their role in intercellular communication among leukocytes. However, interleukins are produced by many other body cells, and their functions are not limited to coordinating leukocyte interactions. Chemokines are a subset of cytokines primarily acting by attracting cells to sites of infection or inflammation [23,25]. Among 41 known interleukins, two—Interleukin-8 (IL-8) and Interleukin-16 (IL-16)—are classified as “interleukins with chemokine activity” [25].

IL-16, initially recognized as a “lymphocyte chemoattractant factor” for CD4-positive cells in 1982, is a multifunctional protein involved in immune response regulation, cell migration, and cell cycle control [26,27,28]. It can be synthesized by various cell types, including CD4 and CD8 T lymphocytes, eosinophils, macrophages, dendritic cells, fibroblasts, mast cells, B lymphocytes, and bronchial epithelium [27,29,30,31]. While CD4 is the primary receptor for IL-16, certain cells can respond to IL-16 through alternative receptors like CD9, for example, mastocytes or lung cancer cells [32], as well as via CD4- and CD9-independent mechanisms [33]. IL-16 attracts immunocompetent cells to sites of inflammation and can stimulate the expression of further pro-inflammatory cytokines such as TNF-α, IL-1β, IL-6, IL-8, and IL-15 [34,35].

The human IL-16 gene, located at chromosome 15q26.3, consists of 153,000 base pairs, 7 exons, and 6 introns, encoding two precursor molecules derived by alternative splicing [26,27,28,31]. The smaller isoform (Pro-IL-16) has 636 amino acids and is expressed in immunocompetent cells, while the larger 1244 amino acid isoform (n-Pro-IL-6) is identified in neuronal cells [26,27,28,31]. Mature IL-16, a 121-amino-acid product, is generated by caspase-3 cleaving the C-terminal domain of either precursor [29,31,32]. Cleaved IL-16 is secreted as a ligand for CD4 with chemoattractant, growth factor, and differentiation factor capabilities, while Pro-IL-16 translocates to the nucleus, acting as a transcriptional repressor of the cell cycle [28,32]. In addition to its role in inflammation, neuronal IL-16 (NIL-16) induces the upregulation of transcription factors, enhances neurite outgrowth, and interacts with neurotransmitter receptors and several ion channel proteins [33,36]. Initially identified in the cerebellum and hippocampus, NIL-16 has recently been identified in osteoarthritic cartilage, suggesting its further peripheral role [37]. Pro-IL-16 contains three PDZ (postsynaptic density protein, disclarge, and zonulin-1) domains for multimerization and a CcN motif for nuclear localization [27,29,38]. The larger neuronal variant has two additional PDZ domains interacting with neuronal ion channels [32,36,38]. PDZ domains bind to protein carboxyl termini or dimerize with other PDZ domains, facilitating IL-16 multimerization into homotetramers [31]. Caspase-3 cleaves IL-16 between PDZ2 and PDZ3 [31,32].

IL-16’s regulatory role in inflammation has led to its study in autoimmune diseases such as asthma [30], inflammatory bowel disease [39], multiple sclerosis [40], systemic lupus erythematosus (SLE) [41,42], and rheumatoid arthritis (RA) [42,43]. It is also implicated in diseases where inflammation modulates disease progression, including viral infections [31,44], depression [45], and cardiovascular diseases [31,46]. Both Pro-IL-16 and secreted IL-16 are relevant to carcinogenesis [32]. Within the tumor microenvironment, IL-16 can attract CD4+ T cells and other immune cells to the tumor site, promoting both pro-tumorigenic and antitumorigenic effects. The production of IL-16 correlates with the onset and progression of gastrointestinal tumors, breast cancer, cutaneous T cell lymphoma (CTCL), and multiple myeloma (MM) [32,46]. The mechanisms by which IL-16 promotes cancer growth vary. In CTCL, a sequence mutation in pro-IL-16 reduces p27KIP1 levels, enhancing cell proliferation; in MM, overexpression of secreted IL-16 induces plasma cell proliferation; and in breast cancer, increased IL-16 recruits CD4+ pro-tumor macrophages [32].

In gynecology, IL-16 is implicated in endometriosis [35,47,48,49,50,51], ovarian physiology [52], and ovarian [53,54] and cervical [55] carcinogenesis. Elevated IL-16 levels in the peritoneal fluid of women with endometriosis, especially in advanced stages, suggest its role in sustaining inflammatory responses in the peritoneal cavity [47]. The study by Zhang et al. demonstrated the pivotal role of pyroptotic T cell-derived active IL-16 as a central cytokine instigating inflammation associated with endometriosis [35]. Cells immunopositive for IL-16 were detected both in ovarian surface epithelium as well as in ovarian stroma [52]. A study using ovarian tissues from menopausal women and patients with HGSOC revealed increased IL-16 expression in the ovarian stroma during menopause [52]. An inverse relationship between IL-16 expression and levels of miR-125a-5p, an IL-16 gene regulator, was observed during ovarian aging, influenced by persistently high levels of FSH [52]. Complementary research using a laying hen model of spontaneous OC demonstrated that IL-16, known for its proangiogenic properties, is associated with OC development and tumor-associated neoangiogenesis [53].

SNPs have been related to structural and functional changes of IL-16 [46,50,55]. Four most commonly studied SNPs within the IL-16 gene are rs4778889, rs11556218, rs4072111, and rs1131445.

rs4778889 (T > C) is located in the promoter region, −295 bp upstream from the transcription start site at the GATA-3 (transcription factor) binding site [56]. The T allele reduces promoter activity compared to the C allele, increasing asthma risk [56]. Conversely, the C allele (TC/CC genotypes) is linked to a higher risk of renal cell carcinoma [57,58], nasopharyngeal cancers [59], and non-cardia gastric cancer [60]. Beyond oncology, the C allele is also associated with endometriosis [48,51] and SLE [41].

rs11556218 (T > G) is a missense mutation in exon 6, causing a substitution of asparagine by lysine in the PDZ2 domain of both pro-IL-16 (Asn446Lys) and n-Pro-IL-16 (Asn1147Lys) [50]. This SNP alters the structural configuration of the PDZ2 domain and disrupts protein–protein recognition by Pro-IL-16 [50]. It is the most studied IL-16 SNP, associated with several benign and malignant conditions. The TG/GG genotypes are linked to higher IL-16 levels [34,59]. The presence of the G allele and G-containing genotypes are strong predictors for developing lung cancer [61], oral cancer [62], nasopharyngeal carcinoma [59,63], gastric cancer [46,60,64,65], colorectal cancer [64], osteosarcoma [34], as well as endometriosis [49,50], coronary artery disease [66,67], ischemic stroke [68], SLE [41], Alzheimer’s disease [69], osteoporosis [70], RA [71], type 2 diabetes mellitus [72,73], and periodontitis [74].

rs4072111 (C > T) is a missense mutation in exon 6, causing a proline to serine change in the nPDZ2 domain of nPro-IL-16 (Pro434Ser) [50]. The T allele has been associated with increased risk of Parkinson’s disease [75], Alzheimer’s [76] disease, and SLE [41], with mixed findings for endometriosis [49,50] and gastric cancer [62,77].

rs1131445 (T > C) affects the miR-135b binding site within the 3′-untranslated region (3′UTR). miRNAs participate in carcinogenesis via post-transcriptional regulation of oncogenes and tumor suppressor genes. The T > C change disrupts the suppressive interaction of miR-135b with IL-16 mRNA, leading to upregulation of IL-16 expression [42,55]. The C allele and C-containing genotypes of rs1131445 are linked to increased risk of cervical cancer [55], colorectal cancer [78], RA, and SLE [42]. In women with cervical cancer, the C allele was associated with higher serum IL-16 concentrations [55].

To date, no study has investigated the role of IL-16 SNPs in OC. To address this gap, we examined the relationship between four common IL-16 polymorphisms and OC risk. We additionally analyzed the role of IL-16 SNPs in relation to menopausal status and specific OC subtypes, HGSOC, and EROC.

## 2. Results

**Figure A1:**
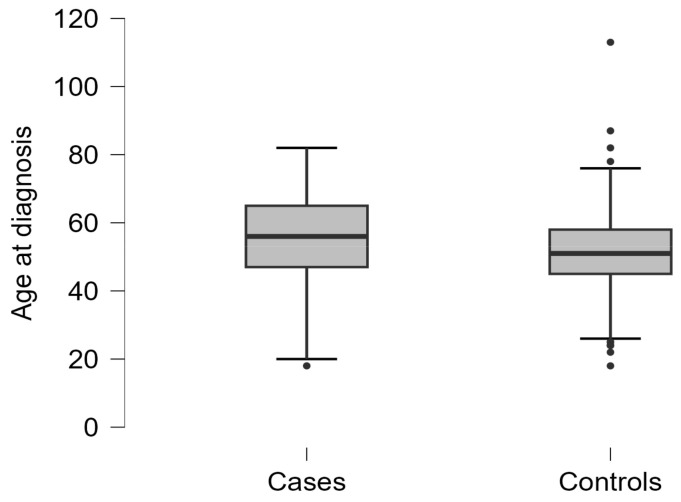
Age at initial diagnosis in cases and controls.

**Figure A2:**
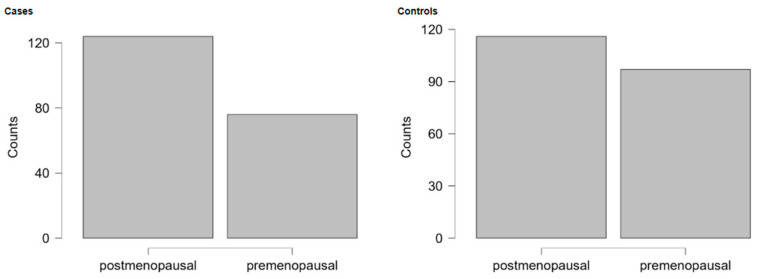
Menopausal status (cutoff point 51 years) of cases and controls.

Within this ethnically homogeneous cohort, 200 women were diagnosed with OC, and 213 were healthy controls. The mean age of cases was 55.8 years (SD 12.3) and the mean age of controls was 51.4 years (SD 13.3), as shown in Figure A1 (Appendix A). The proportion of postmenopausal patients, defined by age ≥ 51 years, did not differ significantly (*p* = 0.12) between cases (62%; *n* = 124/200) and controls (54.5%; *n* = 116/213) (see Figure A2). Regarding OC cases, 169/200 (84.5%) were diagnosed with advanced FIGO stages (IIb-IV). The predominant histological subtype was HGSOC, diagnosed in 73.5% of cases, while 28 out of 200 patients (14%) had endometrioid or clear cell subtypes (collapsed to EROC). The characteristics of the study population are provided in Table 1, while the detailed distribution of histological types is shown in Table A1.

In the control group, the minor allele frequencies (MAF) were 9.2% for rs11556218 (G allele), 13.6% for rs4778889 (C allele), 10.3% for rs4072111 (T allele), and 32.4% for rs1131445 (C allele). The distribution of the respective genotypes did not deviate from Hardy–Weinberg equilibrium for any SNP (see Table 2). These MAFs were comparable to those reported in the gnomAD (Genome Aggregation Database) for the European population, which are 8% for rs11556218 (G allele), 17.8% for rs4778889 (C allele), 10.7% for rs4072111 (T allele), and 34.8% for rs1131445 (C allele) [79].

We are the first to report a strong and significant association between IL-16 rs11556218 (T > G) and OC risk in the general (co-dominant) model (χ^2^ = 28.3, *p* < 0.0001), as well as in the dominant (*p* < 0.0001) and overdominant models (*p* < 0.0001). As presented in Table 2, the G allele was significantly more prevalent in OC cases compared to controls (21.7% vs. 9.2%, OR 2.76, 95% CI 1.84–4.14, *p* < 0.0001). Notably, the significant impact of the G allele on OC risk was observed even in the presence of a single G copy (in GT heterozygotes), as GG homozygotes were rare (2% of cases and no GG homozygotes in controls). Moreover, the significance of the G allele for increased OC risk was observed in both postmenopausal (*p* = 0.001) and premenopausal women (*p* < 0.0001) (see Table 3).

For the entire cohort, the association of IL-16 rs4778889 (T > C) with OC risk was significant in the dominant (*p* = 0.019), overdominant (*p* = 0.033), heterozygote comparison (*p* = 0.027), and allele comparison (C vs. T, OR 1.54, 95% CI 1.06–2.23, *p* = 0.024) models, but narrowly missed significance in the co-dominant model (*p* = 0.057) (Table 2). Notably, the significance of rs4778889 and its C allele was strong in premenopausal women (*p* = 0.012) but diminished after menopause (*p* = 0.29) (Table 3).

In addition, in the co-dominant, recessive, and homozygote models, rs4778889 revealed associations with the EROC subtypes (*p* = 0.002, *p* = 0.006, and *p* = 0.007, respectively) and, in the co-dominant and recessive models, with HGSOS (*p* = 0.036 and *p* = 0.043, respectively). The results in regard to OC subtypes are presented in Table 4 and Table 5.

In contrast, we did not find any significant associations between IL-16 rs4072111 (*p* = 0.814) and IL-16 rs1131445 (*p* = 0.474) and the risk of OC, neither in the entire cohort nor when—as displayed in Table A2—broken down by menopausal status.

The images of the fragment analyzer electropherograms are provided in Supplementary Figures S1–S4.

## 3. Discussion

Inflammatory processes are involved in ovarian pathology, including endometriosis and malignancy [23,24,52,80]. IL-16 expression in tissues and serum correlates with OC development, progression to advanced stages, and peritumoral neoangiogenesis [54]. The high evolutionary conservancy of IL-16 and its CD4 receptor indicates their crucial role in regulating inflammatory responses [26,32]. Thus, even discrete changes in IL-16 structure and function can modulate immune response, influence the tumor microenvironment, and impact cancer cell behavior [23,52].

In this study, we investigated four SNPs within the IL-16 gene: rs4778889 (promoter), rs11556218 (exon 6), rs4072111 (exon 6), and rs1131445 (miRNA binding site in the 3′-UTR). Two of these, rs11556218 (T > G) and rs4778889 (T > C), showed significant associations with OC. The impact of rs11556218 was significant across all age groups and genetic models, while rs4778889 influenced OC risk predominantly in premenopausal women.

The strong association between IL-16 rs11556218 (T > G) and OC risk is attributed to the G allele, which was significantly more prevalent in OC cases (22%) compared to controls (9%). The importance of the G allele was evident even with a single copy (in GT heterozygotes), as GG homozygotes constituted only 2% of cases and were not detectable in controls. Additionally, the association of the G allele with OC risk was highly significant in both postmenopausal and premenopausal women, although stronger in younger patients. While our findings are pioneering for OC, the G allele of rs11556218 is a well-known “disease marker” among IL-16 SNPs. Meta-analyses have confirmed an overall association of this SNP with cancer risk [46,81]. Specifically, individuals carrying the G allele of rs11556218 are at a higher risk for osteosarcoma [34], lung [61], oral [62], nasopharyngeal [59,63], gastric [46,60,64,65], and colorectal [64] cancers. Importantly, the protective effect of not carrying the G allele persisted in TT homozygotes even after correction for risk factors (like smoking, alcohol consumption, and *H. pylori* infection) in gastric cancer [65]. Among benign diseases, most studies confirm a contribution of the G allele to increased risk of cardiovascular diseases [46,66,67,68] and endometriosis [49,50], although results vary across different populations [34,51]. The ultrastructural study by Matalliotakis et al. confirmed that the substitution of asparagine by lysine in the PDZ2 domain of Pro-IL-16 and nPro-IL-16 alters the protein structure and results in an aberrant expression of IL-16 [50]. Higher IL-16 levels, as a functional consequence of rs11556218 TG/GG genotypes, were confirmed in patients with osteosarcoma [34], nasopharyngeal carcinoma [59], and benign conditions like osteoporosis [70]. However, this correlation could not be confirmed in colorectal and gastric carcinomas or healthy controls [64].

A further novel observation is the association of IL-16 rs4778889 (T > C) with OC risk, significant in all but one model for the entire cohort. Notably, the C allele’s association with OC risk was stronger in premenopausal women and fell below the significance threshold after menopause. The next finding specifically observed for rs4778889 was its association with histological subtypes HGSOC and EROC.

These observations provoke several reflections. For both SNPs, rs11556218 and rs4778889, the significance of the results increased in the group of premenopausal patients, indicating an increased relevance of IL-16-mediated processes for ovarian carcinogenesis during the reproductive years. As age increases, immune status shifts, chronic inflammation rises, and the accumulation of mutagens, toxins, and DNA damage occurs, accompanied by impaired repair mechanisms, stem cell exhaustion, and senescence [80,82]. These factors contribute to the rising cancer incidence, including OC, which has been termed an “Inflamm-Aging” condition [80]. Menopause, regardless of the age at which it occurs, signifies fundamental changes in hormonal and immunological homeostasis [52]. Ramirez et al. measured IL-16 expression, macrophage frequency (a source of IL-16), and miR-125a-5p expression (a regulator of the IL-16 gene) in ovarian tissues from women before menopause, at early menopause, and at late menopause. IL-16-expressing cell frequencies were significantly higher in the ovarian stroma of women at early and late menopause compared to premenopausal women. Additionally, miR-125a-5p expression significantly decreased as IL-16 expression increased with aging [52]. The effects of SNPs can vary depending on menopausal status. In our previous study concerning IL-8 polymorphisms, we observed an association of three out of four investigated SNPs—rs4073 (A > T), rs2227306 (C > T), and rs2227543 (C > T)—with OC risk limited to postmenopausal women, emphasizing the role of compensatory mechanisms active in the premenopause [83]. The opposite effects we observed in the present study regarding IL-16 SNPs are especially interesting from a clinical point of view. A younger age of manifestation is the typical hallmark of cancers with a genetic background [84,85]. OC is often missed in younger patients due to the dynamic ultrasound appearance of active ovaries and the escape of early stages from biomarker-based diagnostic strategies [12]. Identifying individuals at risk in this group could enhance personalized detection and treatment strategies.

In the present IL-16 study, we observed associations between rs4778889 and both HGSOC and EROC subtypes. If these results could be confirmed in larger cohorts, rs4778889 might be revealed as a genetic marker of particularly aggressive cancers that occur at a younger age and present with HGSOC morphology. When interpreting the genetic background of EROC, it is difficult to determine whether the particular SNP indicates an association with preexisting endometriosis or a predisposition to the cancerous transformation of endometriosis. Notably, in our IL-8 study, we demonstrated a unique association of the T allele of rs1126647 IL-8 with EROC subtypes [83]. Other studies have shown an association between SNPs involved in the pathogenesis of endometriosis and HGSOC or clear cell OC (the latter belonging to EROC), but not with the endometrioid EROC subtype [86]. The complexity of OC pathology shows that specific OC risk factors can play different roles in women with and without endometriosis [87]. Moreover, endometriosis is not an obligatory precursor for EROC subtypes, and both EROC subtypes can develop through distinct pathways; while clear cell OC may arise from pre-existing endometriotic lesions, endometrioid OC may originate from ovarian Mullerian metaplasia [7]. In this context, we consider it a shortcoming of the study that we did not know the endometriosis history of individual patients.

In contrast to rs11556218 and rs4778889, we did not find any significant associations between IL-16 rs4072111 and IL-16 rs1131445 and OC cancer susceptibility, either in the entire cohort or when broken down by menopausal status.

Notably, rs4072111 is a SNP within the precursor molecule of neuronal Pro-IL-16. However, the cleavage product of n-Pro-IL-16 is IL-16, identical to that of Pro-IL-16, so the extracellular action of secreted IL-16 remains unchanged, regardless of its source. Associations of the T allele with increased risk have been reported for neurological diseases like Parkinson’s [75] and Alzheimer’s [76], as well as SLE [41], and inconsistently with endometriosis [49,50] or gastric cancer [62,77]. Regarding endometriosis and gastric cancer, these associations are not surprising, as endometriosis is accompanied by dysfunctions of the peripheral [88,89] and central [89] nervous systems, and the gastrointestinal system is rich in neuronal cells (often summarized as an independent enteric nervous system) [90]. However, rs4072111 does not seem to be primarily an oncogenic SNP.

The last investigated SNP, rs1131445, is located in a miRNA binding site of the 3′-UTR region and impairs the interaction of miR-135b with IL-16 3′-UTR [55]. MiRNAs are short (18–30 nucleotides) non-coding RNAs involved in cell development and proliferation, tissue differentiation, and programmed cell death [91]. As negative regulators of gene expression, they play an essential role in carcinogenesis. They typically interact with the 3′ UTR of target mRNAs to induce mRNA degradation and translational repression, but can also interact with other regions to regulate transcription or activate translation [92]. The rs1131445 SNP has been linked to colorectal [78] and cervical [55] cancer, with carriers of the rs1131445 C allele showing higher serum IL-16 levels. Mi et al. demonstrated that rs1131445, located in the miR-135b binding site of the IL-16 3′-UTR, affects IL-16 protein expression by interfering with the suppressive function of miR-135b. This interference is significantly associated with an increased risk of cervical cancer in Asian patients [55]. The association of rs1131445 with OC deserves further investigation, as cell-line studies have shown that overexpression of miR-135b promotes growth, migration, and invasion of OC cells. Additionally, overexpression of miR-135b increased cisplatin resistance in selected OC cell lines [93]. The lack of association of rs1131445 with OC risk in the present study may reflect the heterogeneity of OC in vivo and/or the modulating effects of epigenetic and environmental influences on genetic risk factors.

To summarize, our study, which includes all four commonly investigated IL-16 SNPs, has several strengths. First, we are the first to describe an association between IL-16 SNPs and OC risk. In the case of rs11556218, the association is highly significant and consistent with results obtained in other cancer types. Adding the G allele of rs11556218 to the catalog of genetic OC markers could aid in identifying individuals at risk. Second, the subgroup analysis revealed that the relevance of the IL-16 SNPs is particularly significant in premenopausal patients, a finding of potential clinical importance. Third, the possible association with HGSOC and EROC suggests the need for confirmation in larger cohorts. Finally, based on the MAFs reported for the European (non-Finnish) population, the composition of our study was representative of the European population [79]. The homogeneity of the study cohort is a strength, though it may limit the generalizability of our findings to other populations.

From our point of view, the main shortcoming of our study is the absence of data regarding tissue or circulating IL-16 levels in relation to the investigated SNPs, which limits the insights into the mechanisms underlying the observed associations with OC. Second, while the highly significant results for rs11556218 in our study population are unlikely to change with an increased sample size, it is intriguing to consider whether the impact of rs4778889 on OC risk, restricted to premenopausal women in our study, is due to underpowering or solely biological factors. Third, for evaluating the role of specific SNPs, detailed patient history (such as endometriosis history) would allow for a more nuanced interpretation of the results.

## 4. Materials and Methods

### 4.1. Study Design and Participant Demographics

This case-control study investigated four common SNPs within the IL-16 gene: rs4778889 (−295 T > C), rs4072111 (1300 C > T), rs11556218 (3441 T > G), and rs1131445 (3′-UTR T > C), utilizing the restriction fragment length polymorphism (PCR-RFLP) technique. The study cohort comprised 413 Central European women, including 200 ovarian cancer patients and 213 healthy controls. This population and methodology are identical to our prior study concerning IL-8 gene SNPs [83].

### 4.2. Sample Collection and Ethical Compliance

Blood samples were obtained from the Molecular Oncology Group’s blood bank at the Medical University of Vienna. These samples were collected from 1996 to 2021 from women of Central European descent at the Medical University of Vienna and collaborating European institutions. For the present study, we retrieved blood samples originating from Austria, Poland, Germany, and Belgium. All participants provided written informed consent. The blood bank project (EK-366/2003 and EK1966/2020) and the SNP analysis in OC risk (EK-293/2011) received ethical approval from the Medical University of Vienna. Data were anonymized and managed per good scientific practice. Clinicopathological classifications followed WHO (2014) [94] and FIGO (2013) [95] guidelines. Menopausal status was approximated using the age threshold of 51 years, reflecting regional averages [96,97].

### 4.3. DNA Extraction and Genotyping

Peripheral blood samples collected in EDTA tubes were processed to extract genomic DNA from white blood cells using the QIAamp DNA Blood Mini Kit (QIAGEN, Hilden, Germany, cat. no. 51104). IL-16 polymorphisms were identified by analyzing fragment length polymorphisms of the respective PCR products (PCR-RFLP). Amplicons were generated from 25 ng of genomic DNA as a template in a 25 µL reaction mix, containing 5 pmol of the respective forward and reverse primers (Sigma Aldrich, St. Louis, MI, USA, Table 6) and MangoMix™ (Bioline, London, UK, cat. no. BIO-25034) providing MangoTaq™ DNA polymerase, MgCl_2_, and dNTPs. Amplification proceeded with an initial hot start at 95 °C for 5 min, with 45 cycles starting with a 30 s denaturation at 95 °C, followed by a 30 s annealing at the temperature indicated in Table 6, and a 60 s extension at 72 °C. Following a final extension step at 72 °C for 7 min, the PCR products were digested with the respective restriction endonuclease (all provided by New England Biolabs, Ipswich, MA, USA, cat. nos. R0584S (Ahd I), R0529S (BsmA I), R0111S (Nde I), and R0531S (BsaA I)) under conditions specified in Table 6. The restriction fragments were then separated by capillary electrophoresis using the Fragment Analyzer™ Automated CE System (Advanced Analytical, Parkersburg, WV, USA) and the dsDNA 905 Reagent Kit (Agilent, Santa Clara, CA, USA, cat. no. DNF-905). The size of the fragments was determined with PROSize^®^ 3.0 software version 3.0.1.6 (Advanced Analytical Technologies, Orangeburg, NY, USA).

### 4.4. Statistical Analysis

Data analysis was conducted using JASP statistical software v.0.17.3 for Windows [98] and the VassarStats Website for Statistical Computation [99]. Differences in genotype and allele frequencies between patients and controls were evaluated using the Chi-squared (χ^2^) test (with Yates correction for continuity if the numbers in any of the table cells were less than 5) and Fisher’s exact test. To assess the impact of each SNP on OC risk, odds ratios (OR) with 95% confidence intervals (CIs) were calculated. Hardy–Weinberg equilibrium (HWE) in the control group was tested for all four SNPs using the goodness-of-fit χ^2^ test. Age differences between groups were analyzed using the Student’s *t*-test, with a two-sided *p*-value ≤ 0.05 considered statistically significant.

The association between OC risk or traits and IL-16 SNPs was explored using various genetic models, where “A” represents the major allele and “a” represents the minor allele [100,101]:

- Co-dominant Model (General Test of Association): AA versus Aa versus aa.
- Dominant Model: (Aa + aa) versus AA.
- Recessive Model: aa versus (AA + Aa).
- Overdominant Model (Heterozygote Superiority): Aa versus (AA + aa).
- Heterozygote Comparison Model: Aa versus AA.
- Homozygote Comparison Model: AA versus aa.
- Allelic/Multiplicative Model (Allelic Frequency): a versus A.

## 5. Conclusions

The G allele and G-containing genotypes of rs11556218 represent a potential novel genetic marker for elevated risk of OC in European women. The associations of rs11556218 and rs4778889 were particularly pronounced in premenopausal women. Our results can contribute to better identification of individuals at risk and to the development of individualized diagnostic and treatment strategies. Future studies with larger cohorts are warranted to confirm and extend our findings.

## Figures and Tables

**Table 1:** Study population characteristics.

Parameter		Cases	Controls	*p*
Sample size		200	213	
Mean age at diagnosis (years)		55.85 (SD 12.3, IQR 47–65)	51.44 (SD 13.3, IQR 45–58)	<0.001
Menopausal status	Premenopausal	76 (38%)	97 (45.5%)	0.12
	Postmenopausal	124 (62%)	116 (54.5%)	
				
High-grade serous OC	HGSOC	147 (73.5%)		
	Non-HGSOC	46 (23%)		
	N/a	7 (3.5%)		
				
EROC	EROC	28 (14%)		
	Non-EROC	165 (82.5%)		
	N/a	7 (3.5%)		
				
FIGO stage	Early	23 (11.5%)		
	Advanced	169 (84.5%)		
	N/a	8 (4%)		

N/a: non-available for analysis (e.g., only grading available or stage missing), SD—standard deviation, IQR—interquartile range (25–75%), EROC—endometriosis-related OC.

**Table 2:** Genotype and allele distribution of rs11556218, rs4778889, rs4072111, and rs1131445.

Model	Genotype	Controls	Cases	OR (95% CI)	*P* ^Fi^	χ^2^	*P* ^Chi^
**rs11556218 (T > G)**, *p*HWE = 0.141							
Co-dominant	TT	174 (81.7%)	117 (58.5%)	1.00 (Ref.)		28.3 (df = 2)	**<0.0001**
Heterozygote	GT	39 (18.3%)	79 (39.5%)	3.01 (1.92–4.72)	**<0.0001**	24.06	**<0.0001**
Homozygote	GG	0 (0%)	4 (2%)	∞ (NaN–∞)	**0.027**	1.62	0.057
Dominant	GG + GT	39 (18.3%)	83 (41.5%)	3.17 (2.02–4.95)	**<0.0001**	26.65	**<0.0001**
(GG + GT vs. TT)	TT	174 (81.7%)	117 (58.5%)				
Recessive	GG	0 (0%)	4 (2%)	∞ (NaN–∞)	**0.054**	2.47	0.116
GG vs. GT + TT	GT + TT	213 (100%)	196 (98%)				
Overdominant	GT	39 (18.3%)	79 (39.5%)	2.91 (1.86–4.56)	**<0.0001**	22.69	**<0.0001**
(GT vs. TT + GG)	TT + GG	174 (81.7%)	121 (60.5%)				
Allele frequency	G	39 (9.2%)	87 (21.7%)	2.76 (1.84–4.14)	**<0.0001**	25.32	**<0.0001**
(G vs. T), MAF = 0.09	T	387 (90.8%)	313 (78.3%)				
**rs4778889 (T > C)**, *p*HWE = 0.256							
Co-dominant	TT	157 (73.7%)	126 (63%)	1.00 (Ref.)		5.72 (df = 2)	0.057
Heterozygote	CT	54 (25.4%)	70 (35%)	1.62 (1.06–2.47)	**0.031**	4.91	**0.027**
Homozygote	CC	2 (0.9%)	4 (2%)	2.49 (0.45–13.83)	0.414	0.44	0.507
Dominant	CC + CT	56 (26.3%)	74 (37%)	1.65 (1.08–2.5)	**0.020**	5.48	**0.019**
CC + CT vs. TT	TT	157 (73.7%)	126 (63%)				
Recessive	CC	2 (0.9%)	4 (2%)	2.15 (0.39–11.89)	0.436	0.24	0.625
CC vs. CT + TT	CT + TT	211 (99.1%)	196 (98%)				
Overdominant	CT	54 (25.4%)	70 (35%)	1.59 (1.04–2.42)	**0.041**	4.57	**0.033**
CT vs. CC + TT	CC + TT	159 (74.6%)	130 (65%)				
Allele frequency	C	58 (13.6%)	78 (19.5%)	1.54 (1.06–2.23)	**0.024**	5.19	**0.023**
C vs. T, MAF = 0.14	T	368 (86.4%)	322 (80.5%)				
**rs4072111 (C > T)**, *p*HWE = 0.59							
Co-dominant	CC	172 (80.8%)	166 (83%)	1.00 (Ref.)		0.412	0.814
Heterozygote	CT	38 (17.8%)	32 (16%)	0.87 (0.52–1.46)	0.694	0.27	0.603
Homozygote	TT	3 (1.4%)	2 (1%)	0.69 (0.11–4.19)	1	<0.001	1
Dominant	TT + CT	41 (19.2%)	34 (17%)	0.86 (0.52–1.42)	0.610	0.35	0.554
TT + CT vs. CC	CC	172 (80.8%)	166 (83%)				
Recessive	TT	3 (1.4%)	2 (1%)	0.71 (0.12–4.28)	1	<0.001	1
TT vs. CT + CC	CT + CC	210 (98.6%)	198 (99%)				
Overdominant	CT	38 (17.8%)	32 (16%)	0.88 (0.52–1.47)	0.694	0.25	0.617
CT vs. TT + CC	TT + CC	175 (82.2%)	168 (84%)				
Allele frequency	C	382 (89.7%)	364 (91%)	0.86 (0.54–1.36)	0.557	0.42	0.517
T vs. C, MAF = 0.10	T	44 (10.3%)	36 (9%)				
**rs1131445 (T > C)**, *p*HWE = 0.254							
Co-dominant	TT	101 (47.4%)	83 (41.5%)	1.00 (Ref.)		1.49 (df = 2)	0.474
Heterozygote	TC	86 (40.4%)	91 (45.5%)	1.06 (0.57–1.96)	0.876	0.03	0.862
Homozygote	CC	26 (12.2%)	26 (13%)	1.22 (0.66–2.25)	0.637	0.39	0.532
Dominant	CC + CT	112 (52.6%)	117 (58.5%)	1.27 (0.86–1.88)	0.236	1.46	0.227
CC + CT vs. TT	TT	101 (47.4%)	83 (41.5%)				
Recessive	CC	26 (12.2%)	26 (13%)	1.07 (0.60–1.92)	0.882	0.06	0.806
CC vs. TC + TT	TC + TT	187 (87.8%)	174 (87%)				
Overdominant	TC	86 (40.4%)	91 (45.5%)	1.23 (0.83–1.82)	0.32	1.11	0.292
TC vs. TT + CC	TT + CC	127 (59.6%)	109 (54.5%)				
Allele frequency	T	288 (67.6%)	257 (64.25%)	1.16 (0.87–1.55)	0.34	1.03	0.310
C vs. T, MAF = 0.32	C	138 (32.4%)	143 (35.75%)				

*p*HWE—*p* value for the Hardy–Weinberg Equilibrium, MAF: minor allele frequency, *P* ^Fi^—*p* value in Fisher’s exact test, *P* ^Chi^—*p* value in Chi squared test (for df = 1 or df = 2). Significant *p*-values are in bold.

**Table 3:** Genotype and allele distribution of rs11556218 and rs4778889 broken down by menopausal status.

Model	Genotype	Controls	Cases	OR (95% CI)	*P* ^Fi^	χ^2^	*P* ^Chi^
**rs11556218 (T > G)**							
**Postmenopausal**							
Co-dominant	TT	95 (81.9%)	78 (62.9%)	1.00 (Ref.)		11.56	**0.003**
Heterozygote	GT	21 (18.1%)	44 (35.5%)	2.55 (1.4–4.65)	**0.002**	9.66	**0.002**
Homozygote	GG	0 (0%)	2 (1.6%)	∞ (NaN–∞)	0.208		
Dominant	GG + GT	21 (18.1%)	46 (37.1%)	2.67 (1.47–4.85)	**0.001**	10.74	**0.001**
GG + GT vs. TT	TT	95 (81.9%)	78 (62.9%)				
Recessive	GG	0 (0%)	2 (1.6%)	∞ (NaN–∞)	0.498	0.44	0.507
GG vs. GT + TT	GT + TT	116 (100%)	122 (98.4%)				
Overdominant	GT	21 (18.1%)	44 (35.5%)	2.49 (1.37–4.53)	**0.003**	9.17	**0.002**
GT vs. TT + GG	TT + GG	95 (81.9%)	80 (64.5%)				
Allele frequency	G	21 (9.1%)	48 (19.4%)	2.41 (1.39–4.17)	**0.002**	10.34	**0.001**
G vs. T	T	211 (90.9%)	200 (80.6%)				
**Premenopausal**							
Co-dominant	TT	79 (81.4 %)	39 (51.3%)	1.00 (Ref.)		18.74	**<0.0001**
Heterozygote	GT	18 (18.6%)	35 (46.1%)	3.94 (1.98–7.82)	**<0.0001**	16.21	**<0.0001**
Homozygote	GG	0 (0%)	2 (2.6%)	∞ (NaN–∞)	0.115		
Dominant	GG + GT	18 (18.6%)	37 (48.7%)	4.16 (2.11–8.23)	**<0.0001**	17.84	**<0.0001**
GG + GT vs. TT	TT	79 (81.4 %)	39 (51.3%)				
Recessive	GG	0 (0%)	2 (2.6%)	∞ (NaN–∞)	0.192	0.79	0.373
GG vs. GT + TT	GT + TT	97 (100%)	74 (97.4%)				
Overdominant	GT	18 (18.6%)	35 (46.1%)	3.75 (1.89–7.41)	**<0.0001**	15.16	**<0.0001**
GT vs. TT + GG	TT + GG	79 (81.4%)	41 (53.9%)				
Allele frequency	G	18 (9.3%)	39 (25.7%)	3.37 (1.84–6.19)	**<0.0001**	16.62	**<0.0001**
G vs. T	T	176 (90.7%)	113 (74.3%)				
**rs4778889 (T > C)**							
**Postmenopausal**							
Co-dominant	TT	91 (78.4%)	87 (70.2%)	1.00 (Ref.)		5.1	0.078
Heterozygote	CT	23 (19.8%)	37 (29.8%)	1.68 (0.93–3.06)	0.101	2.94	0.086
Homozygote	CC	2 (1.7%)	0 (0%)	0 (0–∞)	0.498	0.44	0.507
Dominant	CC + CT	25 (21.6%)	37 (29.8%)	1.55 (0.86–2.78)	0.184	2.15	0.143
CC + CT vs. TT	TT	91 (78.4%)	87 (70.2%)				
Recessive	CC	2 (1.7%)	0 (0%)	0 (0–∞)	0.233	0.57	0.449
CC vs. CT + TT	CT + TT	114 (98.3%)	124 (100%)				
Overdominant	CT	23 (19.8%)	37 (29.8%)	1.72 (0.95–3.12)	0.100	3.2	0.073
CT vs. CC + TT	CC + TT	93 (80.2%)	87 (70.2%)				
Allele frequency	C	27 (11.6%)	37 (14.9%)	1.33 (0.78–2.27)	0.347	1.12	0.29
C vs. T	T	205 (88.4%)	211 (85.1%)				
**Premenopausal**							
Co-dominant	TT	66 (68%)	39 (51.3%)	1.00 (Ref.)		8.58	**0.014**
Heterozygote	CT	31 (32%)	33 (43.4%)	1.80 (0.96–3.38)	0.078	3.38	0.065
Homozygote	CC	0 (0%)	4 (5.3%)	∞ (0–∞)	**0.022**	4.01	**0.045**
Dominant	CC + CT	31 (32%)	37 (48.7%)	2.02 (1.09–3.75)	**0.029**	5	**0.025**
CC + CT vs. TT	TT	66 (68%)	39 (51.3%)				
Recessive	CC	0 (0%)	4 (5.3%)	0 (0–∞)	**0.04**	3.16	0.076
CC vs. CT + TT	CT + TT	97 (100%)	76 (94.7%)				
Overdominant	CT	31 (32%)	33 (43.4%)	1.63 (0.88–3.05)	0.153	2.4	0.121
CT vs. CC + TT	CC + TT	66 (68%)	43 (56.6%)				
Allele frequency	C	31 (16%)	41 (27%)	1.94 (1.15–3.28)	**0.016**	6.25	**0.012**
C vs. T	T	163 (84%)	111 (73%)				

*P* ^Fi^—*p* value in Fisher’s exact test, *P* ^Chi^—*p* value in Chi squared test (for df = 1 or df = 2). Significant *p*-values are in bold.

**Table 4:** Genotypes and allele frequencies between HGSOC and non-HGSOC subtypes (*n* = 193).

Model	Genotype	Non-HGSOC	HGSOC	OR (95% CI)	*P* ^Fi^	χ^2^	*P* ^Chi^
**rs11556218 (T > G)**							
Co-dominant	TT	28 (60.9%)	82 (55.8%)	1.00 (Ref.)		0.4	0.821
Heterozygote	GT	17 (36.9%)	62 (42.2%)	1.25 (0.63–2.48)	0.605	0.39	0.532
Homozygote	GG	1 (2.2%)	3 (2%)	1.02 (0.1–10.25)	1	<0.0001	1
Dominant	GG + GT	18 (39.1%)	65 (44.2%)	1.23 (0.63–2.42)	0.61	0.37	0.543
	TT	28 (60.9%)	82 (55.8%)				
Recessive	GG	1 (2.2%)	3 (2%)	0.94 (0.1–9.24)	1	<0.0001	1
	GT + TT	45 (97.8%)	144 (98%)				
Overdominant	GT	17 (36.9%)	62 (42.2%)	1.24 (0.63–2.46)	0.608	0.39	0.532
	TT + GG	29 (63.1%)	85 (57.8%)				
Allele frequency (G vs. T)	G	19 (20.7%)	68 (23.1%)	1.16 (0.65–2.05)	0.67	0.25	0.617
	T	73 (79.3%)	226 (76.9%)				
**rs4778889 (T > C)**							
Co-dominant	TT	30 (65.2%)	91 (61.9%)	1.00 (Ref.)		6.66	**0.036**
Heterozygote	CT	13 (28.3%)	55 (37.4%)	1.39 (0.67–2.9)	0.47	0.8	0.371
Homozygote	CC	3 (6.5%)	1 (0.7%)	0.11 (0.01–1.1)	0.056	2.77	0.096
Dominant	CC + CT	16 (34.8%)	56 (38.1%)	1.15 (0.58–2.3)	0.73	0.16	0.689
	TT	30 (65.2%)	91 (61.9%)				
Recessive	CC	3 (6.5%)	1 (0.7%)	0.1 (0.01–0.97)	**0.043**	3.36	0.067
	CT + TT	43 (93.5%)	146 (99.3%)				
Overdominant	CT	13 (28.3%)	55 (37.4%)	1.52 (0.74–3.13)	0.292	1.29	0.256
	CC + TT	33 (71.7%)	92 (62.6%)				
Allele frequency (T vs. C)	C	73 (79.3%)	237 (80.6%)	1.08 (0.6–1.94)	0.881	0.07	0.791
	T	19 (20.7%)	57 (19.4%)				
**rs4072111 (C > T)**							
Co-dominant	CC	37 (80.4%)	122 (83%)	1.00 (Ref.)		0.81	0.668
Heterozygote	CT	8 (17.4%)	24 (16.3%)				
Homozygote	TT	1 (2.2%)	1 (0.7%)				
Dominant	TT + CT	9 (19.6%)	25 (17%)	0.84 (0.36–1.96)	0.824	0.16	0.689
	CC	37 (80.4%)	122 (83%)				
Recessive	TT	1 (2.2%)	1 (0.7%)	0.31 (0.02–5.03)	0.421	0.002	0.97
	CT + CC	45 (97.8%)	146 (99.3%)				
Overdominant	CT	8 (17.4%)	24 (16.3%)	0.93 (0.38–2.23)	1	0.03	0.862
	TT + CC	38 (82.6%)	123 (83.7%)				
Allele frequency (T vs. C)	C	82 (89.1%)	268 (91.2%)	1.26 (0.58–2.72)	0.681	0.34	0.559
	T	10 (10.9%)	26 (8.8%)				
**rs1131445 (T > C)**							
Co-dominant	TT	20 (43.5%)	63 (42.9%)	1.00 (Ref.)		0.04	0.982
Heterozygote	TC	20 (43.5%)	66 (44.9%)	1.05 (0.52–2.13)	1	0.02	0.888
Homozygote	CC	6 (13%)	18 (12.2%)	0.95 (0.33–2.73)	1	0.01	0.920
Dominant	CC + CT	26 (56.5%)	84 (57.1%)	1.03 (0.53–2.0)	1	0.01	0.920
	TT	20 (43.5%)	63 (42.9%)				
Recessive	CC	6 (13%)	18 (12.2%)	0.93 (0.35–2.5)	1	0.02	0.888
	TC + TT	40 (87%)	129 (87.8%)				
Overdominant	TC	20 (43.5%)	66 (44.9%)	1.06 (0.54–2.06)	1	0.03	0.862
	TT + CC	26 (56.5%)	81 (55.1%)				
Allele frequency (C vs. T)	T	60 (65.2%)	192 (65.3%)	0.996 (0.61–1.63)	1	0	1
	C	32 (34.8%)	102 (34.7%)				

*P* ^Fi^—*p* value in Fisher’s exact test, *P* ^Chi^—*p* value in Chi squared test (for df = 1 or df = 2). Significant *p*-values are in bold.

**Table 5:** Genotypes and allele frequencies between EROC and non-EROC subtypes (*n* = 193).

Model	Genotype	Non-EROC	EROC	OR (95% CI)	*P* ^Fi^	χ^2^	*P* ^Chi^
**rs11556218 (T > G)**							
Co-dominant	TT	94 (57%)	18 (64.3%)	1.00 (Ref.)		1.04	0.594
Heterozygote	GT	67 (40.6%)	10 (35.7%)	0.78 (0.34–1.79)	0.678	0.34	0.559
Homozygote	GG	4 (2.4%)	0 (0%)	0 (0–∞)	1	0.03	0.865
Dominant	GG + GT	71 (43%)	10 (35.7%)	0.736 (0.32–1.69)	0.538	0.53	0.466
	TT	94 (57%)	18 (64.3%)				
Recessive	GG	4 (2.4%)	0 (0%)	0 (0–∞)	1	0.01	0.908
	GT + TT	161 (97.6%)	28 (100%)				
Overdominant	GT	67 (40.6%)	10 (35.7%)	0.81 (0.35–1.87)	0.681	0.24	0.624
	TT + GG	98 (59.4%)	18 (64.3%)				
Allele frequency (G vs. T)	G	79 (23.4%)	10 (17.9%)	0.71 (0.34–1.48)	0.395	0.84	0.359
	T	259 (76.6%)	46 (82.1%)				
**rs4778889 (T > C)**							
Co-dominant	TT	106 (64.2%)	16 (57.1%)	1.00 (Ref.)		12.06	**0.002**
Heterozygote	CT	58 (35.2%)	9 (32.1%)	1.03 (0.43–2.47)	1	0.03	0.86
Homozygote	CC	1 (0.6%)	3 (10.7%)	2.99 (0.67–5.31)	**0.011**	7.26	**0.007**
Dominant	CC + CT	59 (35.8%)	12 (42.9%)	1.35 (0.6–3.04)	0.527	0.52	0.471
	TT	106 (64.2%)	16 (57.1%)				
Recessive	CC	1 (0.6%)	3 (10.7%)	2.98 (0.68–5.28)	**0.010**	7.59	**0.006**
	CT + TT	164 (99.4%)	25 (89.3%)				
Overdominant	CT	58 (35.2%)	9 (32.1%)	0.87 (0.37–2.06)	0.833	0.1	0.751
	CC + TT	107 (64.8%)	19 (67.9%)				
Allele frequency (T vs. C)	C	60 (18.2%)	15 (26.8%)	1.65 (0.86–3.17)	0.145	2.26	0.133
	T	270 (81.8%)	41 (73.2%)				
**rs4072111 (C > T)**							
Co-dominant	CC	136 (82.4%)	23 (82.1%)	1.00 (Ref.)		2.14	0.344
Heterozygote	CT	28 (17%)	4 (14.3%)	0.84 (0.27–2.63)	1	<0.001	1
Homozygote	TT	1 (0.6%)	1 (3.6%)	5.91 (0.36–97.9)	0.277	0.16	0.687
Dominant	TT + CT	29 (17.6%)	5 (17.9%)	1.02 (0.36–2.9)	1	<0.001	1
	CC	136 (82.4%)	23 (82.1%)				
Recessive	TT	1 (0.6%)	1 (3.6%)	6.07 (0.37–100.04)	0.27	0.18	0.672
	CT + CC	164 (99.4%)	27 (96.4%)				
Overdominant	CT	28 (17%)	4 (14.3%)	0.82 (0.26–2.53)	0.794	0.006	0.938
	TT + CC	137 (83%)	24 (85.7%)				
Allele frequency (T vs. C)	C	300 (90.9%)	50 (89.3%)	0.83 (0.33–2.10)	0.803	0.15	0.698
	T	30 (9.1%)	6 (10.7%)				
**rs1131445 (T > C)**							
Co-dominant	TT	70 (42.4%)	12 (42.9%)	1.00 (Ref.)		1.02	0.601
Heterozygote	TC	76 (46.1%)	11(39.3%)	0.84 (0.35–2.04)	0.823	0.14	0.708
Homozygote	CC	19 (11.5%)	5 (17.9%)	1.535 (0.48–4.9)	0.529	0.17	0.681
Dominant	CC + CT	95 (57.6%)	16 (57.1%)	0.98 (0.44–2.21)	1	0.002	0.966
	TT	70 (42.4%)	12 (42.9%)				
Recessive	CC	19 (11.5%)	5 (17.9%)	1.67 (0.57–4.91)	0.356	0.398	0.528
	TC + TT	146 (88.5%)	23 (82.1%)				
Overdominant	TC	76 (46.1%)	11(39.3%)	0.76 (0.34–1.72)	0.544	0.44	0.505
	TT + CC	89 (53.9%)	17 (60.7%)				
Allele frequency (C vs. T)	T	216 (65.45%)	35 (62.5%)	0.88 (0.49–1.58)	0.762	0.18	0.671
	C	114 (34.55%)	21 (37.5%)				

*P* ^Fi^—*p* value in Fisher’s exact test, *P* ^Chi^—*p* value in Chi squared test (for df = 1 or df = 2). Significant *p*-values are in bold.

**Table 6:** PCR-RFLP of IL-16 SNPs. Primers for amplification, annealing temperature, and restriction enzyme for digestion.

SNP	Location	Primer Sequence	Annealing Temperature	Digestion (Enzyme, Temperature, Duration)	Fragment Size (bp)
rs4778889 (T > C)	Promoter	Forward: 5′-CTCCACACTCAAAGCCTTTTGTTCCTATGA-3′ Reverse: 5′-CCATGTCAAAACGGTAGCCTCAAGC-3′	60 °C	Ahd I 37 °C, 25 min	T: 280 C: 246 + 34
rs4072111 (C > T)	Exon 6	Forward: 5′-CACTGTGATCCCGGTCCAGTC-3′ Reverse: 5′-TTCAGGTACAAACCCAGCCAGC-3′	67 °C	BsmA I 55 °C, 35 min	C: 164 T: 140 + 24
rs11556218 (T > G)	Exon 6	Forward: 5′-GCTCAGGTTCACAGAGTGTTTCCATA-3′ Reverse: 5′-TGTGACAATCACAGCTTGCCTG-3′	60 °C	Nde I 37 °C, 25 min	G: 171 T: 147 + 24
rs1131445 (T > C)	3′UTR	Forward: 5′-GAGATCATTCACTCATACATCTGG-3′ Reverse: 5′-TCATATACACGCTGGTTCCTTCTG-3′	62 °C	BsaA I 37 °C, 25 min	T: 460 C: 300 + 160

3′UTR—3′ untranslated region. rs number (Ref-SNP)—ID for each SNP assigned by dbSNP.

## Data Availability

The data presented in this study are available on reasonable request from the first (R.W.) or the corresponding (E.O.) author.

## Supplementary Materials

The supporting information can be downloaded at: https://www.mdpi.com/article/10.3390/ijms251910272/s1.

## Appendix A

**Table A1:** Distribution of OC histological subtypes.

Histology	*n*	Percent
Serous *	149	74.5
Endometrioid	23	11.5
Mucinous	8	4.0
Clear cell	5	2.5
Undifferentiated	7	3.5
MMMT	1	0.5
Missing or incomplete (e.g., only grading)	7	3.5
**Total**	**200**	**100**

* Serous histology, including HGSOC and LGSOC.

**Table A2:** Genotype and allele distribution of rs 4072111 and rs 1131445 broken down by menopausal status.

Model	Genotype	Controls	Cases	OR (95% CI)	*P* ^Fi^	χ^2^	*P* ^Chi^
**rs4072111 (C > T)**							
Postmenopausal							
Co-dominant	CC	90 (77.6%)	105 (84.7%)	1.00 (Ref.)		2.5	0.287
Heterozygote	CT	23 (19.8%)	18 (14.5%)	0.67 (0.34–1.32)	0.303	1.34	0.247
Homozygote	TT	3 (2.6%)	1 (0.8%)	0.29 (0.03–2.8)	0.341		
Dominant	TT + CT	26 (22.4%)	19 (15.3%)	0.63 (0.33–1.21)	0.187	1.98	0.159
TT + CT vs. CC	CC	90 (77.6%)	105 (84.7%)				
Recessive	TT	3 (2.6%)	1 (0.8%)	0.31(0.03–2.99)	0.356	0.33	0.567
TT vs. CT + CC	CT + CC	113 (97.4%)	123 (99.2%)				
Overdominant	CT	23 (19.8%)	18 (14.5%)	0.69 (0.35–1.35)	0.306	1.19	0.275
CT vs. TT + CC	TT + CC	93 (80.2%)	106 (85.5%)				
Allele frequency	T	29 (12.5%)	20 (8.1%)	0.61 (0.34–1.12)	0.131	2.57	0.108
T vs. C	C	203 (87.5%)	228 (91.9%)				
Premenopausal							
Co-dominant	CC	82 (84.5%)	61 (80.3%)	1.00 (Ref.)		1.59	0.451
Heterozygote	CT	15 (15.5%)	14 (18.4%)	1.25 (0.56–2.79)	0.682	0.31	0.578
Homozygote	TT	0 (0%)	1 (1.3%)	∞ (NaN–∞)	0.431		
Dominant	TT + CT	15 (15.5%)	15 (19.7%)	1.34 (0.61–2.96)	0.545	0.54	0.462
TT + CT vs. CC	CC	82 (84.5%)	61 (80.3%)				
Recessive	TT	0 (0%)	1 (1.3%)	∞ (NaN–∞)	0.439	0.02	0.902
TT vs. CT + CC	CT + CC	97 (100%)	75 (98.7%)				
Overdominant	CT	15 (15.5%)	14 (18.4%)	1.23 (0.55–2.75)	0.683	0.27	0.603
CT vs. TT + CC	TT + CC	82 (84.5%)	62 (81.6%)				
Allele frequency	T	15 (7.7%)	16 (10.5%)	1.40 (0.67–2.94)	0.448	0.82	0.365
T vs. C	C	179 (92.3%)	136 (89.5%)				
**rs1131445 (3′-UTR T > C)**							
Postmenopausal							
Co-dominant	TT	55 (47.4%)	52 (41.9%)	1.00 (Ref.)		0.75	0.689
Heterozygote	TC	46 (39.7%)	55 (44.4%)	1.26 (0.73–2.18)	0.409	0.71	0.399
Homozygote	CC	15 (12.9%)	17 (13.7%)	1.2 (0.54–2.64)	0.691	0.2	0.655
Dominant	CC + CT	61 (52.6%)	72 (58.1%)	1.25 (0.75–2.08)	0.436	0.73	0.393
(DD, Dd) vs. dd	TT	55 (47.4%)	52 (41.9%)				
Recessive	CC	15 (12.9%)	17 (13.7%)	1.07 (0.51–2.26)	1	0.03	0.862
DD vs. (Dd, dd)	TC + TT	101 (87.1%)	107 (86.3%)				
Overdominant	TC	46 (39.7%)	55 (44.4%)	1.21 (0.73–2.03)	0.514	0.54	0.462
	TT + CC	70 (60.4%)	69 (55.6%)				
Allele frequency	T	156 (67.2%)	159 (64.1%)	1.15 (0.79–1.68)	0.501	0.52	0.471
C vs. T	C	76 (32.8%)	89 (35.9%)				
Premenopausal							
Co-dominant	TT	46 (47.4%)	31 (40.8%)	1.00 (Ref.)		0.8	0.672
Heterozygote	TC	40 (41.2%)	36 (47.4%)	1.34 (0.70–2.53)	0.417	0.79	0.374
Homozygote	CC	11 (11.3%)	9 (11.8%)	1.21 (0.45–3.27)	0.800	0.15	0.699
Dominant	CC + CT	51 (52.6%)	45 (59.2%)	1.31 (0.71–2.40)	0.442	0.76	0.383
(DD, Dd) vs. dd	TT	46 (47.4%)	31 (40.8%)				
Recessive	CC	11 (11.3%)	9 (11.8%)	1.05 (0.41–2.68)	1	0.01	0.92
DD vs. (Dd, dd)	TC + TT	86 (88.7%)	67 (88.2%)				
Overdominant	TC	40 (41.2%)	36 (47.4%)	1.28 (0.7–2.35)	0.444	0.65	0.42
	TT + CC	57 (58.8%)	40 (52.6%)				
Allele frequency	T	132 (68%)	98 (64.5%)	1.17 (0.75–1.84)	0.494	0.49	0.484
C vs. T	C	62 (32%)	54 (35.5%)				

*P* ^Fi^—*p* value in Fisher’s exact test, *P* ^Chi^—*p* value in Chi squared test (for df = 1 or df = 2).
