# Supplementary material for: Association of the Single Nucleotide Polymorphisms rs11556218, rs4778889, rs4072111, and rs1131445 of the Interleukin-16 Gene with Ovarian Cancer
Source: Int J Mol Sci. 2024 Sep 24;25(19):10272. doi: 10.3390/ijms251910272 (PMC11477281; doi:10.3390/ijms251910272)
Supplement: Supplementary file 1 [file ijms-25-10272-s001.zip › ijms-3212252-supplementary.pdf]

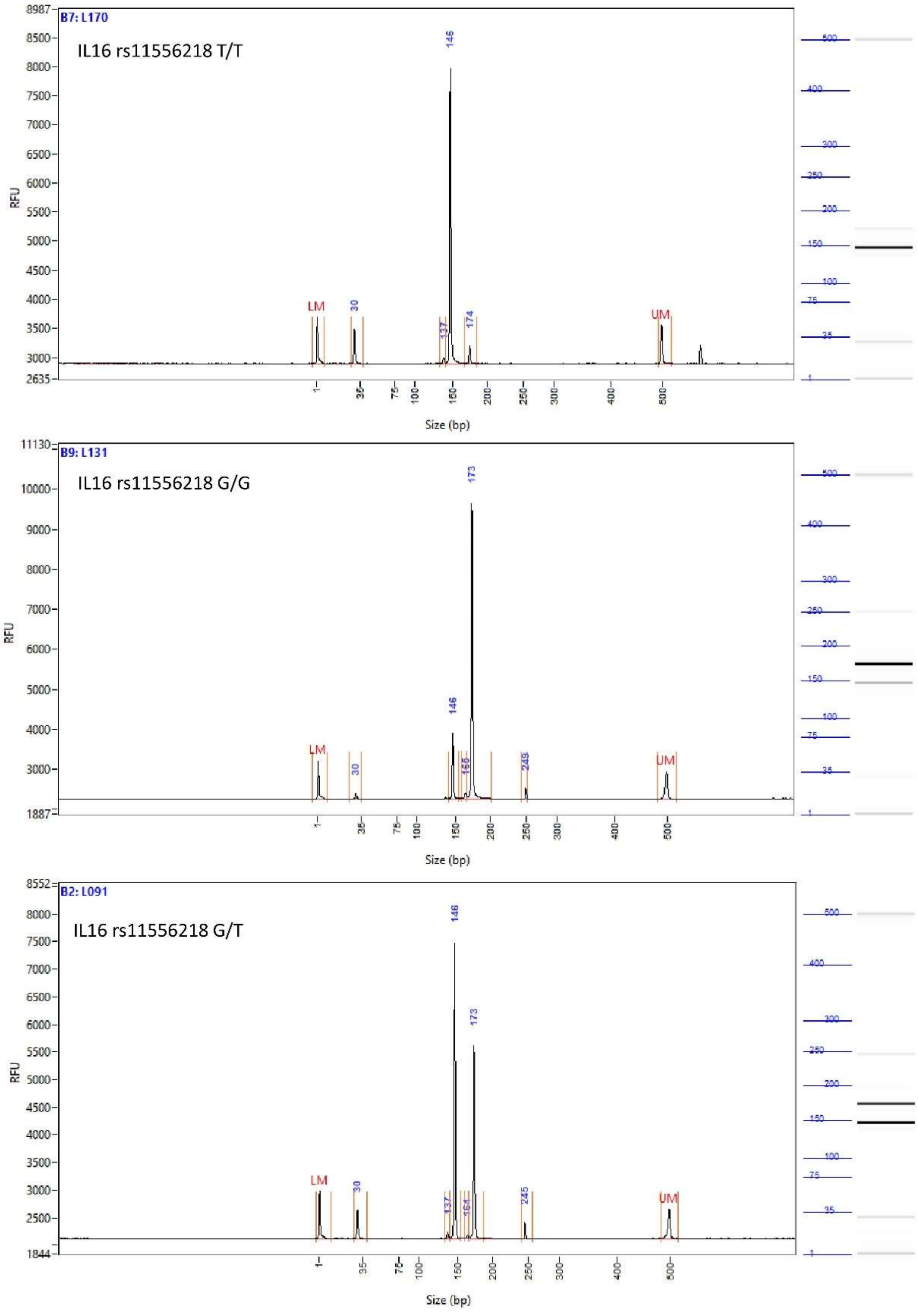

Figure S1. Image of the fragment analyzer electropherograms for representative samples with IL16 rs11556218 genotypes T/T (**top** panel), G/G (**middle** panel), and G/T (**bottom** panel). Nde I restriction fragments were separated by capillary electrophoresis using the Fragment Analyzer™ Automated CE System and the DNF-905 dsDNA Kit. The presence of a fragment at 171bp suggests the presence of the G/G genotype, while fragments at 147 bp and 24 bp suggest the presence of the T/T genotype. With the G/T genotype, all fragments (171 bp, 147 bp, and 24 bp) are present. UM upper marker, LM lower marker.

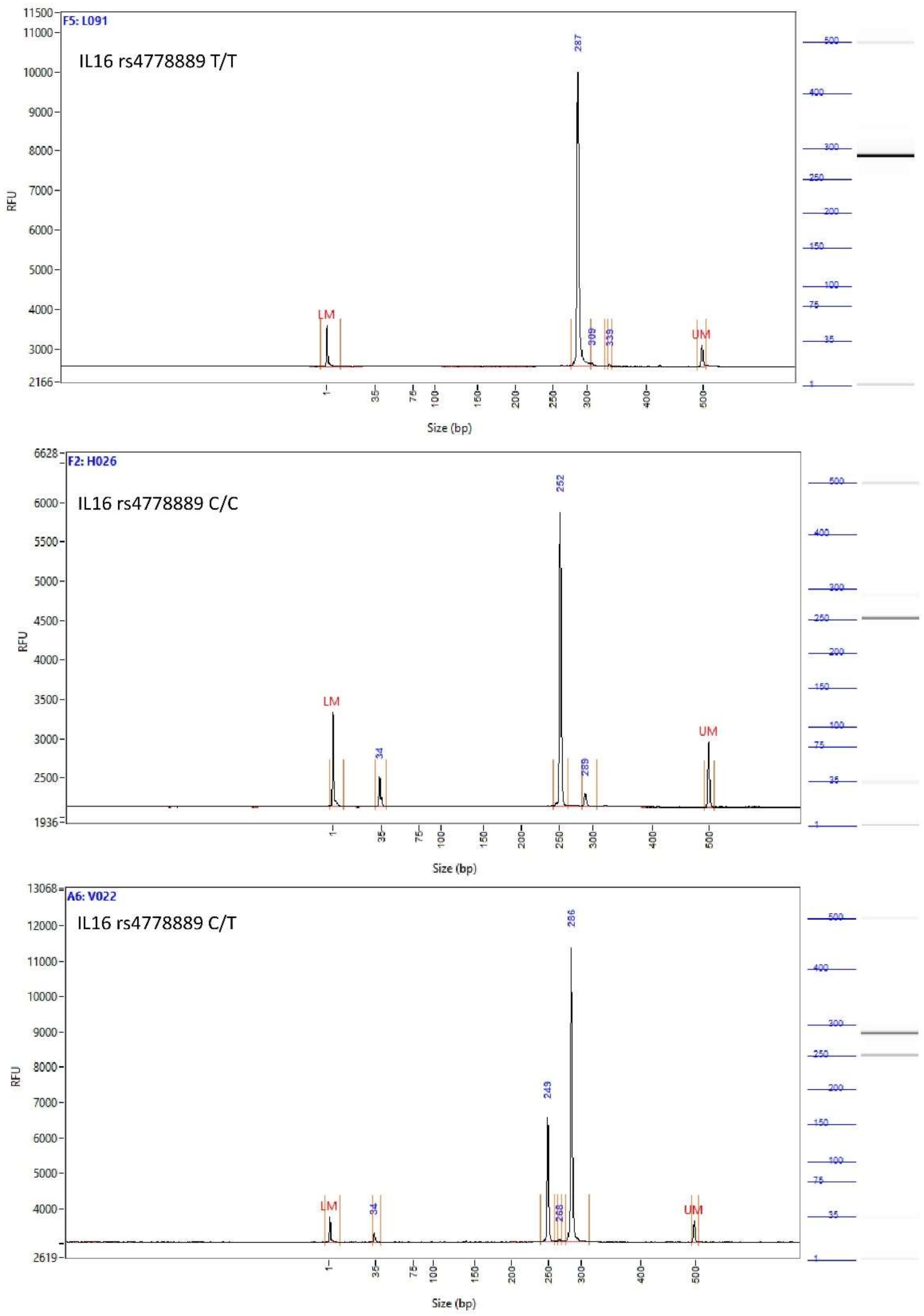

Figure S2. Image of the fragment analyzer electropherograms for representative samples with IL16 rs4778889 genotypes T/T (**top** panel), C/C (**middle** panel), and C/T (**bottom** panel). Ahd I restriction fragments were separated by capillary electrophoresis using the Fragment Analyzer™ Automated CE System and the DNF-905 dsDNA Kit. The presence of a fragment at 280 bp suggests the presence of the T/T genotype, while fragments at 246 bp and 34 bp suggest the presence of the C/C genotype. With the C/T genotype, all fragments (280 bp, 246 bp, and 34 bp) are present. UM upper marker, LM lower marker.

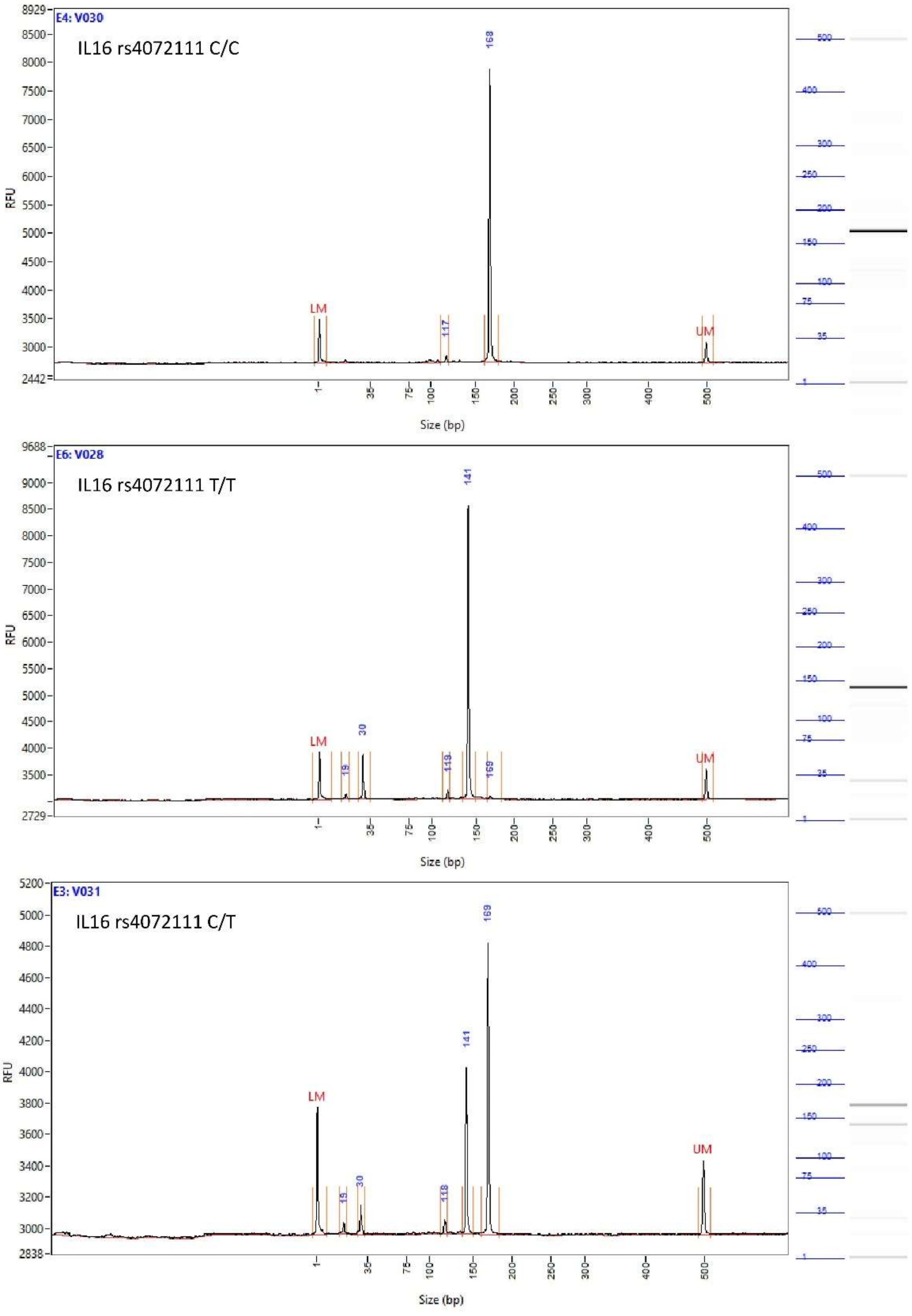

Figure S3. Image of the fragment analyzer electropherograms for representative samples with IL16 rs4072111 genotypes C/C (**top** panel), T/T (**middle** panel), and C/T (**bottom** panel). BsmA I restriction fragments were separated by capillary electrophoresis using the Fragment Analyzer™ Automated CE System and the DNF-905 dsDNA Kit. The presence of a fragment at 164bp suggests the presence of the C/C genotype, while fragments at 140 bp and 24 bp suggest the presence of the T/T genotype. With the C/T genotype, all fragments (164 bp, 140 bp, and 24 bp) are present. UM upper marker, LM lower marker.

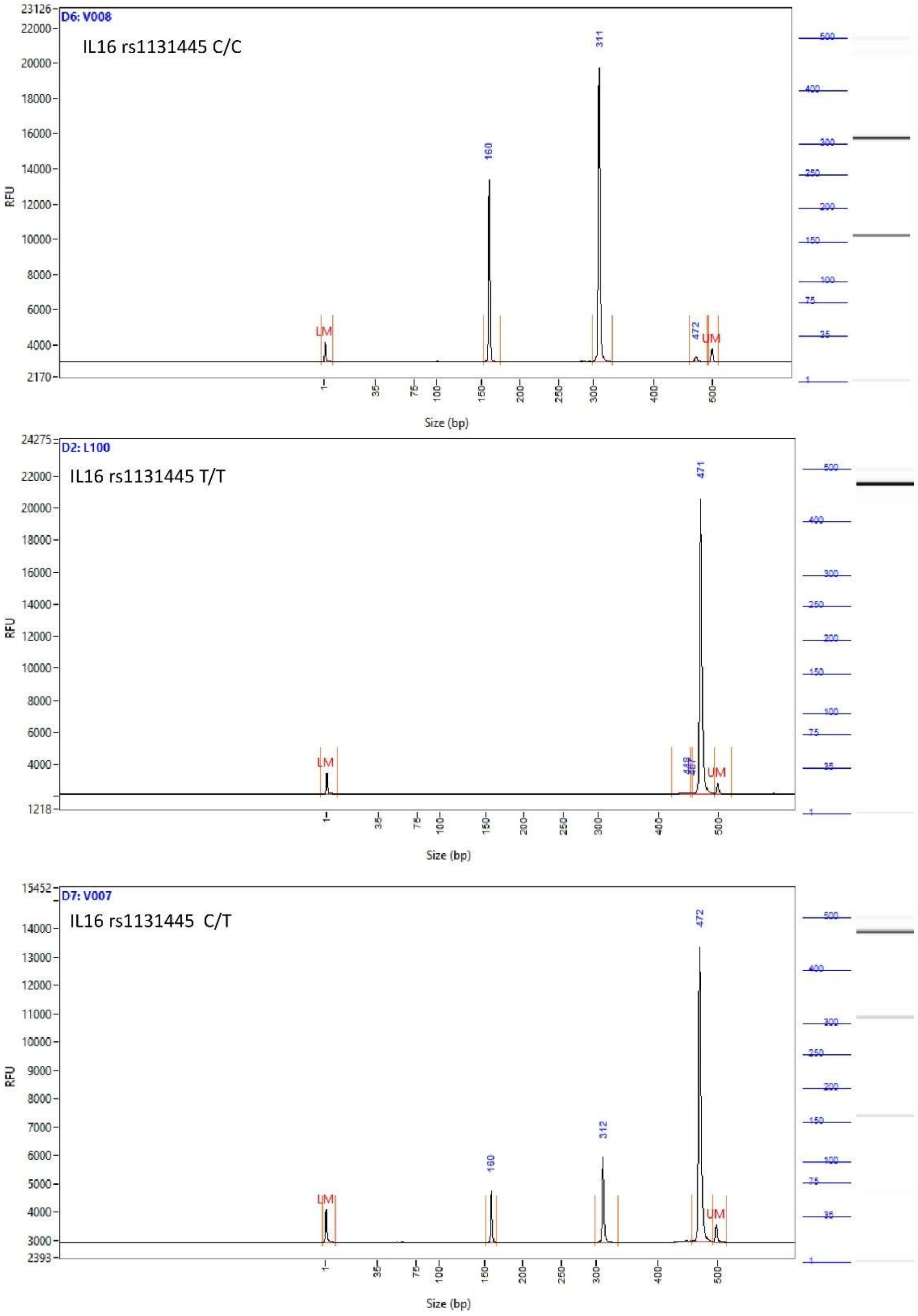

Figure S4. Image of the fragment analyzer electropherograms for representative samples with IL16 rs1131445 genotypes C/C (**top** panel), T/T (**middle** panel), and C/T (**bottom** panel). BsaA I restriction fragments were separated by capillary electrophoresis using the Fragment Analyzer™ Automated CE System and the DNF-905 dsDNA Kit. The presence of a fragment at 460 bp suggests the presence of the T/T genotype, while fragments at 300 bp and 160 bp suggest the presence of the C/C genotype. With the C/T genotype, all fragments (460 bp, 300 bp, and 160 bp) are present. UM upper marker, LM lower marker.
